# The performance of anthropometric measures to predict diabetes mellitus and hypertension among adults in Jordan

**DOI:** 10.1186/s12889-019-7801-2

**Published:** 2019-10-29

**Authors:** Yousef Khader, Anwar Batieha, Hashem Jaddou, Mohammed El-Khateeb, Kamel Ajlouni

**Affiliations:** 1 Department of Public Health, Jordan University of Science and Technology, P.O.Box 3030, Irbid, 22110 Jordan; 2 The National Center for Diabetes, Endocrinology and Genetics, The Jordan University, Amman, Jordan

**Keywords:** Anthropometric measures, Diabetes mellitus, Hypertension, Receiver operating characteristics

## Abstract

**Objectives:**

This study aimed to evaluate and compare the abilities of waist circumference (WC), body mass index (BMI), hip circumference (HC), waist-to-hip ratio (WHR) and waist-to-height ratio (WHtR) to predict recently and previously diagnosed diabetes and hypertension and assess their appropriate cut-off values among Jordanian adults.

**Methods:**

Data from the 2017 cardiovascular risk factors survey were analyzed to achieve the study objective. The survey collected extensive data from a national population-based sample of Jordanian residents. A structured questionnaire was used to collect sociodemographic variables and clinical data. Blood samples were taken for biochemical measurements. Anthropometric characteristics were measured by the same team of trained field researchers.

**Results:**

This study included a total of 1193 men and 2863 women. Their age ranged from 18 to 90 year with a mean (SD) of 43.8 (14.2) year. WHtR performed better than other anthropometric measures and had a good ability (AUC > 0.80) among women and fair ability among men to predict newly diagnosed diabetes and previously diagnosed diabetes and hypertension. The appropriate cut-off points for anthropometric measures among women were 92 cm form WC, 104 cm for HC, 30 Kg/m^2^ for BMI, 0.85 for WHR, and 0.60 for WHtR. For men, the appropriate cut-off points were 100 cm for WC, 104 cm for HC, 27 Kg/m^2^ for BMI, 0.93 for WHR, and 0.57 for WHtR.

**Conclusion:**

WHtR performed better than other anthropometric measures in predicting diabetes and hypertension among adult population in Jordan. We recommend WHtR as a measure of choice with a cut-off value of 0.6 for women and 0.57 for men to predict diabetes and hypertension among Jordanians.

## Introduction

The global burden of non-communicable diseases (NCDs) is immense and increasing. NCDs accounted for 70% of the estimated 56.4 million deaths worldwide in 2015, with almost 80% of these deaths are occurring in low- and middle-income countries [1]. In the Eastern Mediterranean Region (EMR), 57% of deaths in the region are attributed to NCDs [2]. Unhealthy diets, physical inactivity, and obesity are the main underlying risk factors of NCDs in the EMR [3]. In Jordan, the overall age-standardized prevalence rate of diabetes increased from 13.0% in 1994 to 23.7% in 2017 [4]. In 2017, the age-standardized prevalence of hypertension in Jordan was 33.8% among men and 29.4% among women [5].

Central obesity is a common cardiometabolic risk factor. Anthropometric measures including waist circumference (WC), body mass index (BMI), hip circumference (HC), waist-to-hip ratio (WHR) and waist-to-height ratio (WHtR) have been commonly used to predict the risk of diabetes and hypertension. BMI and WC are the most commonly used anthropometric measures for prediction of cardiometabolic risk factors [6]. However, BMI does not provide information on body fat distribution and WC does not take into account intraindividual and ethnic differences in lean body mass, body shape and height [6]. WHtR demonstrated higher ability than other anthropometric measures in predicting cardiometabolic abnormalities by taking into account both central fat deposition and intraindividual differences in height [7].

Considerable controversy still exists as to which measure most accurately defines body fat distribution. Studies from different countries and ethnicities in the world showed that anthropometric measures have different predictive powers for diabetes and hypertension [8–24]. Therefore, the predictive power of anthropometric measures and their appropriate cut-off points should be established for different ethnicities. This study aimed to evaluate and compare the abilities of WC, BMI, WHR, HC and WHtR to predict recently and previously diagnosed diabetes and hypertension and assess their appropriate cut-off values among Jordanian adults.

## Methods

### Study design and sampling

A multipurpose national household survey was conducted among Jordanian adults over a period of four months in the year 2017. The detailed study design, methods, and procedures were described in previous publications [4, 5]. In brief, a multistage sampling technique was used to select a nationally representative sample from the population of Jordan. A village/city was selected from each of the 12 governorates of Jordan. The study procedures took place in the main health center in the selected villages/cities. The research team visited the households in the catchment area of the health center to invite adults to report to the center fasting in a given day after explaining the study for them. Subjects were asked not to take their medications in that day and to bring the medications with them to the health center. Subjects aged ≥18 years were eligible for inclusion in the study.

### Data collection

Data were collected using a structured validated and pilot tested questionnaire by trained interviewers. The questionnaire was the same one that has been used in 2009 survey. The questionnaire included questions to assess the sociodemographic variables and clinical characteristics of participants, including self-reported diagnosis and treatment of diabetes and hypertension. Three blood samples were drawn from a cannula inserted into the antecubital vein and used for the different laboratory measurements. Tubes containing sodium fluoride potassium oxalate were used for glucose measurement. Samples were centrifuged within 1 h at the survey site, and transferred by separate labeled tubes in ice boxes to the central laboratory of the National Center of Diabetes, Endocrinology, and Genetics in Amman, Jordan. All biochemical measurements were carried out by the same team of laboratory technicians using the same method throughout the study period. Fasting plasma glucose was measured by the glucose oxidase method, using a Cobas Analyzer (Roche).

### Anthropometric measurements

Single measurement was taken for each anthropometric measures. Weight was measured, while subjects minimally clothed without shoes using digital scales (seca). Height was measured using a portable stadiometer (SECA 214 portable stadiometer). BMI was calculated as weight in kilograms divided by height in meters squared. WC was measured at the midway between iliac crest and lower rib margin, over light clothing, using unstretchable tape (seca 203), without any pressure to body surface. WHR was calculated as WC divided by hip circumference and WHtR as WC divided by height in centimeter. Measurements were taken by the same team of well-trained persons using the same tools.

### Variable definitions

Recently diagnosed diabetes was defined as having fasting blood sugar ≥126 mg/dl (≥7.0 mmol/l) at the time of the survey with no prior history of diabetes. Recently diagnosed hypertension was defined as having a blood pressure ≥ 140 mmHg systolic and/ or 90 mmHg diastolic at the time of the survey, with no prior history of hypertension. Previously diagnosis of diabetes and hypertension were self-reported by participants.

### Statistical analysis

Data were entered and analyzed using the IBM SPSS, version 20. Data were described using means and percentages. Percentages were compared using chi-square test and difference between means were tested using independent t test. The performance of anthropometric measures was evaluated using receiver operating characteristics (ROC) analyses for each gender [25]. Validity of the test was assessed by calculating area under curve (AUC). The AUC values were classified as: 0.5–0.6 fail, 0.6–0.7 poor, 0.7–0.8 fair, 0.8–0.9 good and 0.9–1.0 excellent [26]. Appropriate cut-off values were defined based on Youden’s J statistic (maximum [sensitivity + specificity - 1]). To further judge the ability of anthropometric measures to predict previously and recently diagnosed diabetes and hypertension, anthropometric measures were dichotomized based on the established cut-off values in this study and tested for their associations using binary logistic regression after adjusting for age effect. All regression analyses were stratified by gender.

We did not adjust for multiple comparisons in analysis because almost all *p*-values were almost close to 0. A *p*-value < 0.05 was considered statistically significant.

## Results

### Participants’ characteristics

This study included a total of 1193 men and 2863 women. Their aged ranged from 18 to 90 years with a mean (SD) of 43.8 (14.2) year. About 27.3% of men and 15.1% of women were previously diagnosed with diabetes, 3.7% of men and 2.2% of women were recently diagnosed with diabetes, 28.2% of men and 21.4% of women were previously diagnosed with hypertension and 27.1% of men and 18.5% of women were recently diagnosed with hypertension. Table 1 shows the socio-demographic, anthropometric, and clinical characteristics of participants according to gender.

**Table 1:** The socio-demographic, anthropometric, and clinical characteristics of participants according to gender

	Women *N* = 2863		Men *N* = 1193		Total	*P*-value
	n	%	n	%	N	
Age (year)						< 0.001
< 50	1966	68.8	648	54.4	2614	
≥ 50	890	31.2	543	45.6	1433	
Marital status						< 0.001
Single	462	16.1	144	12.1	606	
Married	2401	83.9	1049	87.9	3450	
Region						< 0.001
North	922	32.2	390	32.7	1312	
Middle	1295	45.2	471	39.5	1766	
South	646	22.6	332	27.8	978	
Smoking status						< 0.001
None smoker	2628	91.8	592	49.6	3220	
Past smoker	46	1.6	206	17.3	252	
Current smoker	189	6.6	395	33.1	584	
High triglycerides level	1036	36.2	647	54.2	1683	< 0.001
Low HDL	1659	57.9	732	61.4	2391	< 0.001
Previously diagnosed diabetes	433	15.1	326	27.3	759	< 0.001
Recently diagnosed diabetes	64	2.2	44	3.7	108	< 0.001
Previously diagnosed hypertension	614	21.4	336	28.2	950	< 0.001
Recently diagnosed hypertension	529	18.5	323	27.1	852	< 0.001

Table 2 shows the means and the 95% confidence intervals for the anthropometric characteristics for men and women. Women had significantly higher means of BMI, HC and WHtR and lower means of WC and WHR than men.

**Table 2:** The mean and the 95% confidence intervals for the anthropometric characteristics for men and women

	Women *N* = 2827			Men *N* = 1185			*P*-value
Anthropometric measures	Mean	95% Confidence Interval		Mean	95% Confidence Interval		
Waist Circumference (cm)	92.7	92.1	93.4	98.9	98.0	99.8	< 0.001
Hip Circumference (cm)	108.3	107.8	108.8	104.7	104.1	105.4	< 0.001
Body Mass Index (Kg/m^2^)	30.0	29.8	30.2	28.4	28.2	28.7	< 0.001
Waist to Hip Ratio	0.86	0.85	0.86	0.94	0.94	0.95	< 0.001
Waist to Height Ratio	0.59	0.58	0.59	0.57	0.57	0.58	< 0.001

**Fig. 1:**
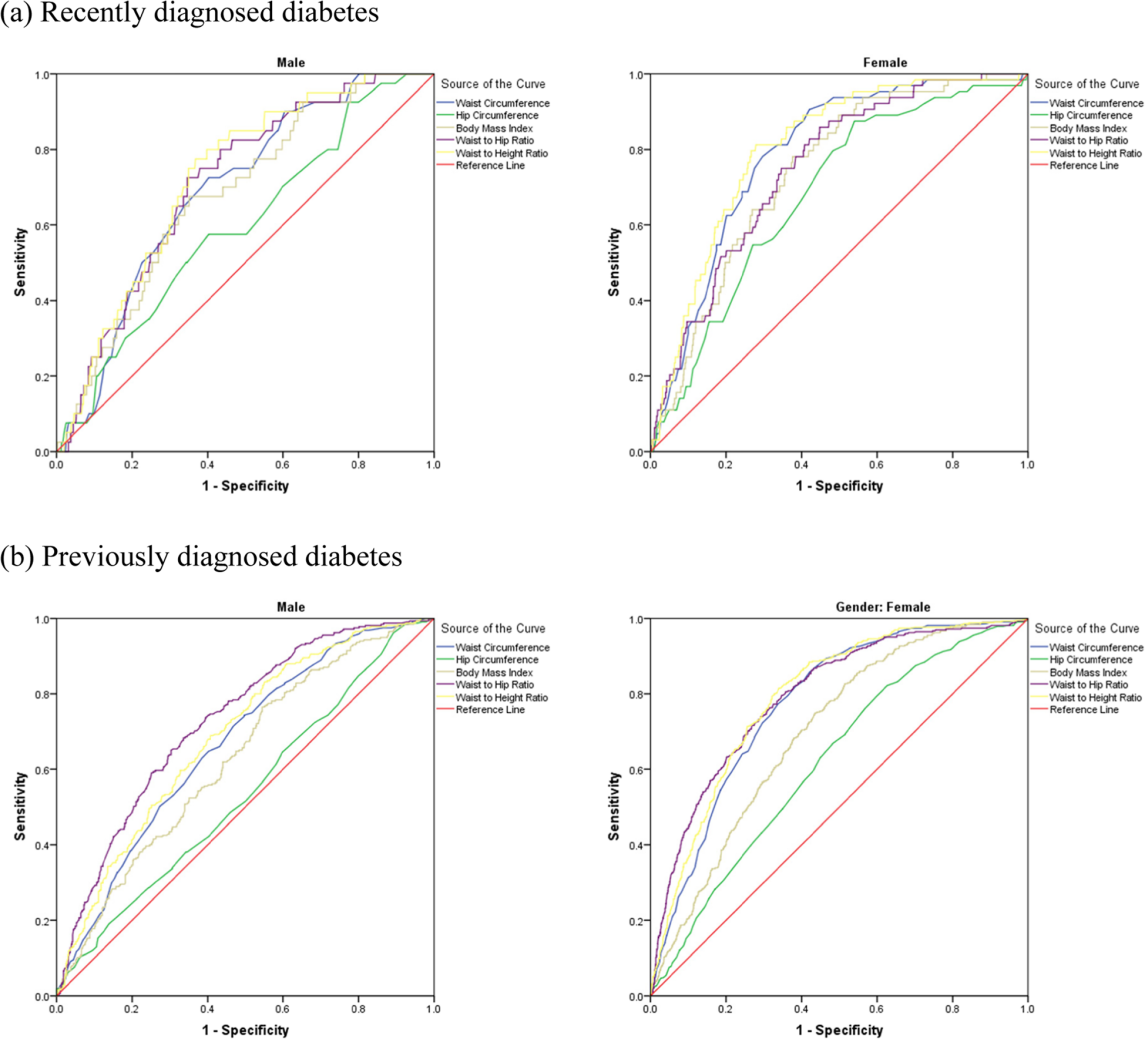
Receiver operating characteristic curve illustrating the ability of anthropometric measures to predict **a** recently diagnosed diabetes and **b** previously diagnosed diabetes according to gender

**Fig. 2:**
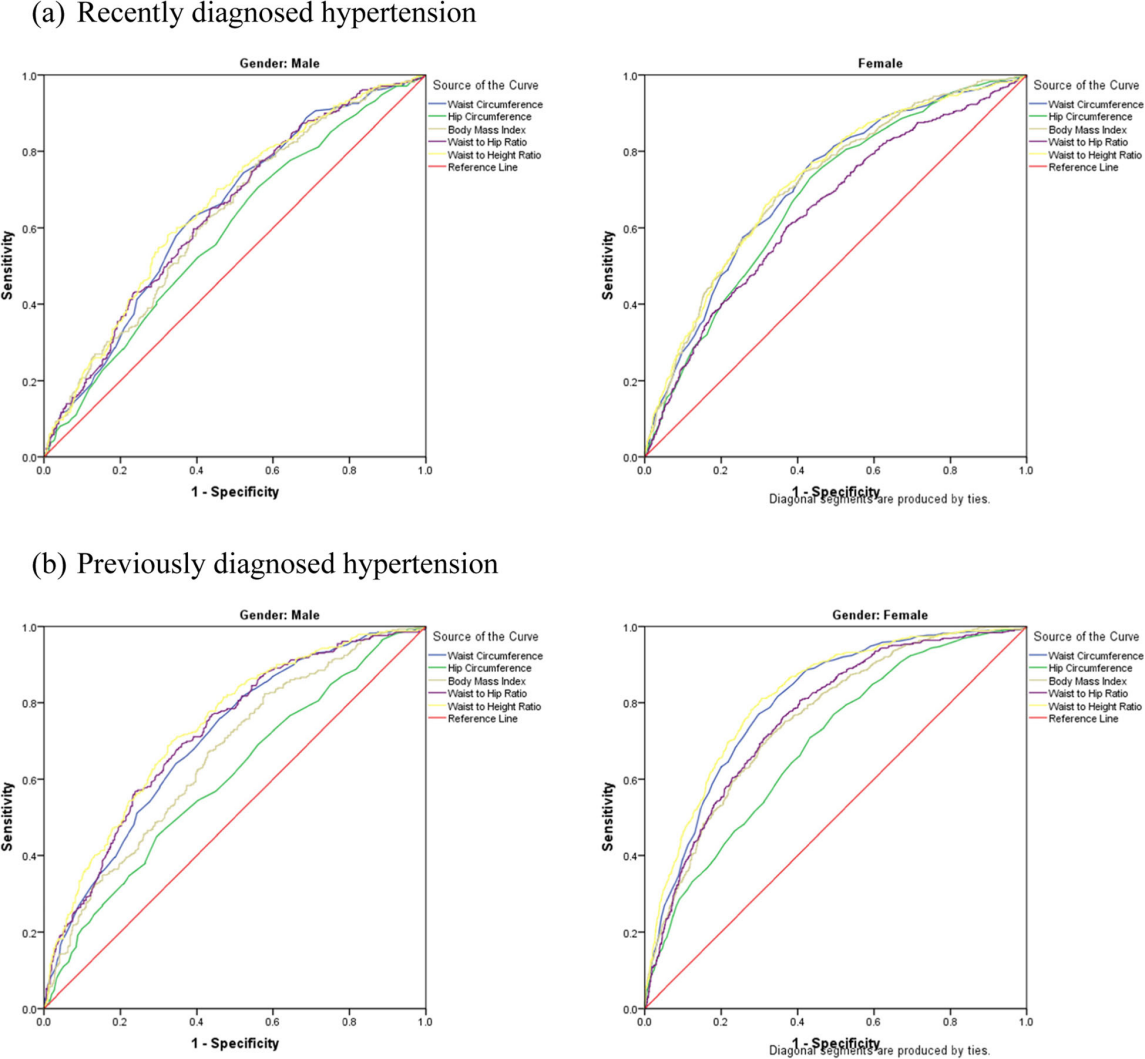
Receiver operating characteristic curve illustrating the ability of anthropometric measures to predict **a** recently diagnosed hypertension and **b** previously diagnosed hypertension according to gender

ROC analysis (Figs. 1 and 2) showed that all anthropometric measures had better performance to predict recently and previously diagnosed diabetes and hypertension among women than that among men. Table 3 shows the area under the ROC curve for men and women. Among women, WHtR performed better than other anthropometric measures and had a good accuracy (AUC > 0.80) to predict incident and prevalent cases of diabetes and prevalent cases of hypertension. It had fair accuracy to predict incident cases of hypertension. Among men, WHtR had fair accuracy and performed better than other anthropometric measures to predict recently and previously diagnosed diabetes and previously diagnosed hypertension. Moreover, WC had a good accuracy to predict recently diagnosed diabetes and previously diagnosed hypertension among women and fair accuracy to predict recently diagnosed diabetes and previously diagnosed hypertension among men. BMI and WHR had fair accuracy and HC had poor accuracy to predict the studied outcomes among women and had poor performance to predict the studied outcomes in men.

**Table 3:** Area under receiver operating characteristic curve illustrating the ability of anthropometric measures to predict recently and previously diagnosed diabetes and hypertension according to gender

	Women			Men		
	AUC	95% confidence interval		AUC	95% confidence interval	
Recently diagnosed diabetes						
Waist Circumference	0.80	0.74	0.83	0.70	0.62	0.76
Hip Circumference	0.68	0.62	0.74	0.60	0.51	0.68
Body Mass Index	0.74	0.69	0.79	0.68	0.60	0.76
Waist to Hip Ratio	0.75	0.70	0.80	0.69	0.64	0.78
Waist to Height Ratio	0.81	0.76	0.85	0.72	0.66	0.79
Previously diagnosed diabetes						
Waist Circumference	0.78	0.75	0.76	0.67	0.63	0.65
Hip Circumference	0.62	0.59	0.61	0.54	0.50	0.52
Body Mass Index	0.70	0.67	0.69	0.63	0.59	0.61
Waist to Hip Ratio	0.80	0.77	0.78	0.69	0.65	0.67
Waist to Height Ratio	0.81	0.77	0.78	0.73	0.70	0.71
Recently diagnosed hypertension						
Waist Circumference	0.71	0.69	0.74	0.64	0.60	0.68
Hip Circumference	0.68	0.66	0.71	0.59	0.55	0.63
Body Mass Index	0.71	0.69	0.74	0.63	0.59	0.67
Waist to Hip Ratio	0.71	0.69	0.74	0.64	0.60	0.68
Waist to Height Ratio	0.72	0.69	0.74	0.66	0.62	0.69
Previously diagnosed hypertension						
Waist Circumference	0.80	0.78	0.82	0.70	0.67	0.74
Hip Circumference	0.69	0.67	0.72	0.60	0.56	0.64
Body Mass Index	0.76	0.74	0.78	0.66	0.62	0.70
Waist to Hip Ratio	0.76	0.74	0.78	0.96	0.68	0.75
Waist to Height Ratio	0.82	0.80	0.83	0.73	0.70	0.77

Youden’s J statistic was used to capture the performance of anthropometric measures and define appropriate cut-off values. The appropriate cut-off points for anthropometric measures among women were 92 cm for WC, 104 cm for HC, 30 Kg/m^2^ for BMI, 0.85 for WHR, and 0.60 for WHtR. For men, the appropriate cut-off points were 100 cm for WC, 104 cm for HC, 27 Kg/m^2^ for BMI, 0.93 for WHR, and 0.57 for WHtR (Table 4).

**Table 4:** Appropriate anthropometric measures cut-off values for predicting previously and recently diagnosed diabetes and hypertension and their values of sensitivity and specificity

	Women			Men		
	Cut-off value	Sensitivity	Specificity	Cut-off value	Sensitivity	Specificity
Waist Circumference						
Recently diagnosed diabetes	92.0	81%	66%	100.0	65%	66%
Previously diagnosed diabetes	92.0	82%	62%	100.0	71%	53%
Recently diagnosed hypertension	90.0	76%	58%	100.0		
Previously diagnosed hypertension	92.0	82%	65%	100.0	64%	65%
Hip Circumference						
Recently diagnosed diabetes	104.0	88%	46%	104.0	58%	60%
Previously diagnosed diabetes	100.0	82%	37%	100.0	94%	55%
Recently diagnosed hypertension	104.0	73%	57%	104.0	54%	60%
Previously diagnosed hypertension	104.0	77%	51%	102.0	62%	51%
Body Mass Index						
Recently diagnosed diabetes	30.0	56%	77%	27.0	63%	68%
Previously diagnosed diabetes	28.0	77%	54%	27.0	77%	50%
Recently diagnosed hypertension	30.0	68%	66%	27.0	77%	50%
Previously diagnosed hypertension	30.0	72%	67%	27.0	82%	50%
Waist to Hip Ratio						
Recently diagnosed diabetes	0.85	75%	65%	0.93	75%	65%
Previously diagnosed diabetes	0.85	76%	68%	0.93	65%	69%
Recently diagnosed hypertension	0.85	61%	62%	0.93	64%	57%
Previously diagnosed hypertension	0.85	72%	68%	0.93	69%	64%
Waist to Height Ratio						
Recently diagnosed diabetes	0.60	84%	64%	0.57	80%	73%
Previously diagnosed diabetes	0.60	83%	63%	0.57	68%	60%
Recently diagnosed hypertension	0.60	71%	63%	0.57	70%	67%
Previously diagnosed hypertension	0.60	81%	69%	0.57	71%	65%

All anthropometric measures were dichotomized using the established cut-off values and tested for their associations with the studied outcomes after adjusting for age effect (Table 5). All dichotomized measures were significantly associated with increased odds of recently and previously diagnosed diabetes and hypertension, except HC that did not show significant association with recently and previously diagnosed diabetes. Among women, WC > 92 cm and WHtR> 0.60 were significantly associated with almost 3 to 5 times increased odds of recently and previously diagnosed diabetes and hypertension. Among men, WC > 100 cm and WHtR> 0.57 were significantly associated with almost 2 to 4 times increased odds of recently and previously diagnosed diabetes and hypertension.

**Table 5:** Age-adjusted associations between anthropometric measures that were dichotomized based on the established cut-off values and previously and recently diagnosed diabetes and hypertension

	Women				Men			
	Odds ratio	95% confidence interval		*p*-value	Odds ratio	95% confidence interval		*p*-value
Recently diagnosed diabetes								
Waist Circumference	5.1	2.3	11.0	< 0.001	3.2	1.6	6.5	0.001
Hip Circumference	3.1	1.5	6.7	0.003	1.6	0.8	3.3	0.157
Body Mass Index	3.3	1.8	6.2	< 0.001	2.7	1.3	5.8	0.011
Waist to Hip Ratio	2.9	1.5	5.4	0.001	2.7	1.3	5.8	0.011
Waist to Height Ratio	5.1	2.4	10.9	< 0.001	4.0	1.8	8.6	0.001
Previously diagnosed diabetes								
Waist Circumference	3.1	2.3	4.2	< 0.001	2.5	1.6	3.2	< 0.001
Hip Circumference	1.4	1.1	1.9	0.019	1.3	0.9	1.7	0.147
Body Mass Index	1.8	1.4	2.4	< 0.001	2.4	1.7	3.3	< 0.001
Waist to Hip Ratio	3.2	2.4	4.3	< 0.001	2.4	1.7	3.3	< 0.001
Waist to Height Ratio	3.3	2.5	4.5	< 0.001	2.5	1.4	3.1	< 0.001
Recently diagnosed hypertension								
Waist Circumference	2.8	1.8	3.1	< 0.001	2.3	1.7	3.1	< 0.001
Hip Circumference	2.6	2.0	3.3	< 0.001	1.9	1.4	2.6	< 0.001
Body Mass Index	2.7	2.2	3.3	< 0.001	2.1	1.6	2.9	< 0.001
Waist to Hip Ratio	1.5	1.2	1.9	< 0.001	1.7	1.2	2.3	< 0.001
Waist to Height Ratio	2.8	2.0	3.2	< 0.001	2.3	1.7	3.1	< 0.001
Previously diagnosed hypertension								
Waist Circumference	3.3	2.5	4.4	< 0.001	2.6	1.9	3.6	< 0.001
Hip Circumference	2.4	1.8	3.1	< 0.001	1.9	1.3	2.6	< 0.001
Body Mass Index	2.8	2.2	3.6	< 0.001	2.5	1.7	3.5	< 0.001
Waist to Hip Ratio	2.1	1.6	2.6	< 0.001	2.0	1.4	2.8	< 0.001
Waist to Height Ratio	3.4	2.6	4.4	< 0.001	2.6	1.9	3.7	< 0.001

## Discussion

The findings of previous studies that compared the predictive power of anthropometric measures to predict cardiometabolic conditions are contradicting. Our study demonstrated a higher ability for WHtR to predict diabetes and hypertension among Jordanian adult men and women compared to other measures. This finding is consistent with the findings of other studies that showed WHtR a better predictor compared to other measures among women [8–10] and men [11] of different populations including a meta-analysis [12] of ten studies and a systematic review of 13 studies which demonstrated superiority of WHtR over other measures [13]. WHtR has been argued to be superior to a single measure of WC by taking into account intraindividual and ethnic differences in height [6]. However, there was a lot of inconsistency regarding the different anthropometric measures in predicting diabetes and hypertension. WHR was reported to be a better predictor in a number of countries [14–17]. Also, WC was reported to show superiority over other anthropometric measures in the prediction of type 2 diabetes in British women [18], U.S. men [19], German women [11], and Indian men and women [20].

Regarding BMI, systematic reviews [6, 21] encompassing Asian and Caucasian populations have consistently reported the inferior utility of BMI in identifying undiagnosed diabetes as compared with abdominal indices.

On the other hand, other studies demonstrated that BMI, WHR, WC, and WHtR had similar predictive powers for the risk of type 2 diabetes [24]. WHR, WC, and WHTR performed similarly well in Bangladeshi women [23]. A meta-analysis of 32 studies [24] showed that BMI, WHR, and WC had similar associations with incident diabetes.

Possible reasons for variation between studies might be due to ethnic and racial differences and differences in body composition and fat distribution between different ethnic groups, genders and age groups. Other reasons might include different study designs, different WC measurement protocols or different methods for defining cardiometabolic outcomes.

The appropriate cut-off points for predicting diabetes and hypertension among Jordanian women were 92 cm form WC, 104 cm for HC, 30 Kg/m^2^ for BMI, 0.85 for WHR, and 0.60 for WHtR. For men, the appropriate cut-off points were 100 cm for WC, 104 cm for HC, 27 Kg/m2 for BMI, 0.93 for WHR, and 0.57 for WHtR. Different cut-off values were reported for other populations. Ethnic and racial differences might explain the discrepancy in cut-off values between different studies.

The findings of ROC analysis in this study are supported by findings of the age-adjusted associations of dichotomized anthropometric measures with the outcome variables. WHtR and WC, using the established cut-off values, had strong association with previously and recently diagnosed diabetes and hypertension.

Our study had several strengths including a large national sample. In addition, the anthropometric measures were performed by the same team of field researchers. The main limitation of this study is the lower response rate (40%) in males. This is expected given that the employment rate in men in Jordan is much higher than that in women. An important limitation to consider when interpreting the findings is the possibility of measurement error (e.g. we measured the WC over light clothing). Another limitation is that the predictive ability of these anthropometric measures is correlated or associated with cardiovascular disease risk factors but not with cardiovascular disease events itself. So further studies are needed to correlate these measures with actual events.

## Conclusions

WHtR performed the best in predicting diabetes and hypertension among adult population in Jordan. We recommend WHtR as a measure of choice with a cut-off value of 0.6 for women and 0.57 for men to predict diabetes and hypertension among Jordanians. Moreover, we recommend that WHtR should be measured routinely in health system and used as one of the indicators for the risk assessment of diabetes and hypertension.

## Data Availability

The datasets used and/or analysed during the current study are available from the corresponding author on reasonable request.

## Abbreviations

AUC: Area under curve
BMI: Body mass index
EMR: Eastern Mediterranean Region
HC: Hip circumference
NCDs: Non-communicable diseases
ROC: Receiver operating characteristics
WC: Waist circumference
WHR: Waist-to-hip ratio
WHtR: Waist-to-height ratio
